# Acetylated α‐tubulin alleviates injury to the dendritic spines after ischemic stroke in mice

**DOI:** 10.1111/cns.14184

**Published:** 2023-03-25

**Authors:** Chuanyan Yang, Xuezhu Chen, Chenxu Zhang, Xuejiao Lei, Yongling Lu, Yuhai Wang, Hua Feng, Tunan Chen, Yang Yang

**Affiliations:** ^1^ Department of Neurosurgery Southwest Hospital, Third Military Medical University (Army Medical University) Chongqing 400038 China; ^2^ Department of Neurosurgery, the 904^th^ Hospital of PLA School of Medicine of Anhui Medical University Wuxi Jiangsu Province 214044 China; ^3^ Clinical Medical Research Center Southwest Hospital, Third Military Medical University (Army Medical University) Chongqing 400038 China

**Keywords:** acetylated α‐tubulin, dendritic spine, ischemic stroke, microtubule, synapse

## Abstract

**Background and Aim:**

Functional recovery is associated with the preservation of dendritic spines in the penumbra area after stroke. Previous studies found that polymerized microtubules (MTs) serve a crucial role in regulating dendritic spine formation and plasticity. However, the mechanisms that are involved are poorly understood. This study is designed to understand whether the upregulation of acetylated α‐tubulin (α‐Ac‐Tub, a marker for stable, and polymerized MTs) could alleviate injury to the dendritic spines in the penumbra area and motor dysfunction after ischemic stroke.

**Methods:**

Ischemic stroke was mimicked both in an in vivo and in vitro setup using middle cerebral artery occlusion and oxygen–glucose deprivation models. Thy1‐YFP mice were utilized to observe the morphology of the dendritic spines in the penumbra area. MEC17 is the specific acetyltransferase of α‐tubulin. Thy1 Cre^ERT2‐eYFP^ and MEC17^fl/fl^ mice were mated to produce mice with decreased expression of α‐Ac‐Tub in dendritic spines of pyramidal neurons in the cerebral cortex. Moreover, AAV‐PHP.B‐DIO‐MEC17 virus and tubastatin A (TBA) were injected into Thy1 Cre^ERT2‐eYFP^ and Thy1‐YFP mice to increase α‐Ac‐Tub expression. Single‐pellet retrieval, irregular ladder walking, rotarod, and cylinder tests were performed to test the motor function after the ischemic stroke.

**Results:**

α‐Ac‐Tub was colocalized with postsynaptic density 95. Although knockout of MEC17 in the pyramidal neurons did not affect the density of the dendritic spines, it significantly aggravated the injury to them in the penumbra area and motor dysfunction after stroke. However, MEC17 upregulation in the pyramidal neurons and TBA treatment could maintain mature dendritic spine density and alleviate motor dysfunction after stroke.

**Conclusion:**

Our study demonstrated that α‐Ac‐Tub plays a crucial role in the maintenance of the structure and functions of mature dendritic spines. Moreover, α‐Ac‐Tub protected the dendritic spines in the penumbra area and alleviated motor dysfunction after stroke.

## Graphical abstract

α‐Ac‐Tub plays a crucial role in maintaining the structure and function of dendritic spines. Synapse injury leads to the disruption of the neural circuit and neurological dysfunction after stroke. Protection of more dendritic spines in the penumbra area is a promising therapeutic strategy for stroke. α‐Ac‐Tub was colocalized with postsynaptic density protein. Specifically, the knockout of MEC17 (an α‐tubulin acetyltransferase) could aggravate the synapse injury of pyramidal neurons in the penumbra area. However, specific upregulation of MEC17 in the pyramidal neurons and TBA treatment could alleviate synapse injury in the penumbra area and relieve motor dysfunction after stroke.

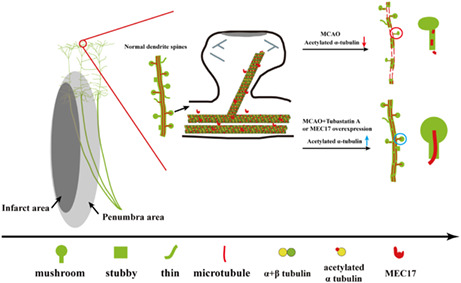

## 1 INTRODUCTION

Ischemic stroke is a global leading cause of permanent disability.^1^ Neurological impairments caused by focal stroke are associated with abnormal synaptic activity, cell death, and disruption of neural circuits.^2^ So far, intensive rehabilitation is the preferred measure to improve motor function in patients after stroke.^3^ However, numerous patients still have significant motor deficits. Mechanically, rehabilitative training promotes improved motor function by enhancing dendritic spines' formation and circuit wiring after focal ischemic damage. The number of dendritic spines formed correlated with the degree of functional recovery after stroke.^4^ However, due to the reduced neural plasticity in the senile population, rehabilitative training is often not highly effective. Moreover, recovery is associated with the preservation of dendritic spines in surviving peri‐infarct regions.^5^ Therefore, an alternative approach is to develop precautionary measures to further avoid injury to the dendritic spines after a stroke.

The dendritic spines are small, specialized, and thin protrusions arising from the neuronal dendrites that are predominantly located at the excitatory synapses.^6^ Increased levels of actin and actin‐associated proteins present in these structures control and regulate the dynamic morphology of spines.^6^ In contrast, as an important type of cytoskeleton, the significance of microtubules (MTs) in the development of dendritic spines and plasticity is largely unknown.^7^ Interestingly, in a previous study, live 3‐D confocal imaging was used to identify MTs in spines, especially in “mature spines, that are mushroom‐shaped. This could be attributed to the retention of GFP‐tubulin signals within the spine head with polymerized MTs.^8^, ^9^ Furthermore, the knockdown of end‐binding protein 3 (EB3) has been shown to significantly reduce the development of spines. Additionally, the stabilization of MTs using taxol was shown to enhance spine formation, whereas their inhibition using nocodazole impaired the process of spine formation. This was in turn attributed to brain‐derived neurotrophic factors. Thus, polymerized MTs play a predominant role in the regulation of the control of the dendritic spine and its plasticity. However, the post‐translational modifications (PTMs) of MT subunits determine the stability of the MT and play critical roles in functional specialization.^10^, ^11^, ^12^ To date, the function of PTMs in dendritic spine formation and their maintenance is largely unknown.

Among the PTMs, acetylation of K40 in α‐tubulin (α‐Ac‐Tub) is the sole PTM to characterize the luminal surface of MTs.^13^, ^14^ In mammals, the levels of α‐Ac‐Tub are regulated by acetylation and deacetylation, which are catalyzed by α‐tubulin acetyltransferase 1 (αTAT1, also known as Mec17) and histone deacetylase 6 (HDAC6), respectively.^15^ Interestingly, α‐Ac‐Tub is the most abundant in polymerized MTs; however, it is absent from dynamic cellular structures.^16^ This is consistent with a previous study that reported that polymerized MTs are present in “mature spines that are mushroom‐shaped.” However, the significance of α‐Ac‐Tub in the formation and maintenance of dendritic spines remains poorly understood. We studied the effect of the upregulation of α‐Ac‐Tub in the preservation of dendritic spines in the penumbra area and the alleviation of motor dysfunction after stroke.

## 2 MATERIALS AND METHODS

### 2.1 Animals

Thy1‐YFP mice (Stock No: 003782, Jackson Laboratory) and Thy1 Cre^ERT2‐eYFP^ mice^17^ (Stock No: 012708, Jackson Laboratory) were received from The Jackson Laboratory. MEC17^fl/fl^ mice were obtained from Cyagen Biosciences Inc. The Thy1 Cre^ERT2‐eYFP^ and MEC17^fl/fl^ mice were mated to produce Thy1 Cre^ERT2‐eYFP^::MEC17^fl/fl^ mice. Tamoxifen (75 mg/kg body weight; 10,540–29‐1, Sigma) was dissolved in corn oil and intragastrically administered once every 24 h for five consecutive days to induce gene recombination in 4‐week‐old Thy1 Cre^ERT2‐eYFP^ mice. Adult mice that weighed approximately 20–25 g and were 8–10 weeks old were used in this study. Pregnant C57/BL6 mice were received from the Experimental Animal Center of Third Military Medical University. Random grouping of the mice was performed in odd/even numbers. Randomization was conducted in odd/even numbers. The mice were maintained in a humidity‐controlled room (with a temperature maintained at 25 ± 1°C, and a 12‐h light/dark cycle) with food and water ad libitum. The results of all the performed experiments were compliant with the Animal Research: Reporting of In Vivo Experiments (ARRIVE) guidelines. The protocols used in this study were subjected to approval from the Laboratory Animal Welfare and Ethics Committee of Third Military Medical University (AMUWEC20210680). The experiments were performed in accordance with the guidelines of the Guide for the Care and Use of Laboratory Animals.

### 2.2 Middle cerebral artery occlusion/reperfusion model of mice and treatment protocol

A mouse model for the middle cerebral artery occlusion/reperfusion model was developed following the procedure of middle cerebral artery occlusion (MCAO) as described in a previous study conducted by our group.^18^ Briefly, anesthesia of the mice was carried out using a 2% mixture of isoflurane and air (1–2 L/min). After making a cut in the midline skin and dissecting the carotid artery, an 8–0 nylon suture that was 2.0 cm silicone coated was introduced carefully into the external carotid artery stump through the internal carotid artery, which was terminated at the middle cerebral artery opening. Ligation was conducted around 120 min before restoration of the cerebral blood flow. A feed‐back‐controlled heating pad was used to retain the body temperature at 37 ± 0.3°C during surgery. Mice were fed ad libitum food and water post‐surgery. After the mice recovered, neurological deficits were graded as described previously using a four‐point scale for neurological deficit severity.^19^ Mice with scores 2–3 were used for the subsequent experiments. The same procedure was performed with sham‐operated mice without the introduction of the suture to the internal carotid artery. Tubastatin A (TBA; SML0044, Sigma) was first dissolved in dimethyl sulfoxide (DMSO) to form a 10 mg/mL stock solution, which was further diluted with saline, 1000 times. The mice were then administered TBA (25 mg/kg, intraperitoneally (i.p.)) or a corresponding equivalent of the vehicle (Veh, 1% DMSO) immediately after MCAO. Additionally, to overexpress MEC17 (NM_001142744), the AAV/PHP.B‐Syn‐flex‐MEC17 (100 μL, tilter 1 × 10^13^ copies/mL; BrainVTA, Wuhan, China) was intravenously injected via the tail vein 3 weeks before the induction of stroke. After 24 h of stroke, the recognizable dendritic spines in the ischemic penumbra area were observed and quantified for comparison in a double‐blind manner.

### 2.3 Primary neuron culture and oxygen–glucose deprivation (OGD)

The pregnant mice at E16.5 were used for primary cortical neuron culture. Post‐isolation, neuronal cultures were maintained described previously.^20^ The fetal brains were removed and the cerebral cortices were dissected to remove the surrounding meninges. The cortices were pooled and subjected to digestion with papain for a duration of 10 min at 37°C. Following centrifugation, the supernatant was filtered using a 50 μm strainer to eliminate chunks of undissociated tissue. Subsequently, following adjustments to the density of the cells (1 × 10^4^ cells in a 75 μL suspension), cortical neuronal cells were plated on poly‐l‐ornithine coated (10 μg/mL for 2 h) 12 mm culture dishes (Sigma‐Aldrich). Finally, the maintenance medium was added to the culture dishes and was replaced with a fresh neural medium every 2 days. The maintenance medium included the following: MEM (51200–038, a volume of 41.25 mL/50 mL, Gibco), 1 M glucose (15023–021, a volume of 1 mL/50 mL, Invitrogen), sodium pyruvate (S8636, a volume of 0.5 mL/50 m, Sigma), HEPES solution (H0887, a volume of 1.25 mL/50 mL, Sigma), N‐2 supplement (17502–048, a volume of 5 mL/50 mL, Invitrogen), and penicillin/streptomycin (15140–122, 0.5 mL/50 mL, Invitrogen).

To establish OGD conditions,^21^ cells mixed with Earle's balanced salt solution (glucose‐free) were allowed to incubate for 2 h in a hypoxic chamber. The chamber was continuously flushed with 95% N_2_ and 5% CO_2_ and maintained at 37°C to achieve a final concentration of 0.5% O_2_. Reoxygenation of the cells was performed using a maintenance medium with 95% air and 5% CO_2_. The cells were then subjected to TBA treatment (1 μM) or vehicle (Veh) for 12 h immediately after OGD.

### 2.4 Immunofluorescence staining

For immunofluorescence staining, sections of the brain or primary cultured neuronal dishes were fixed using paraformaldehyde. Subsequently, a blocking solution was prepared with 10% normal goat serum and 0.5% Trition X‐100. The samples were then incubated in the blocking solution for 2 h at room temperature prior to the staining procedure. Furthermore, respective primary antibodies [rabbit anti‐α‐Ac‐Tub (ab179484, 1:1000, Abcam) and mouse anti‐PSD95 (ab2723, 1:1000, Abcam)] were added to the samples and incubated overnight at 4°C. Subsequently, the sections or dishes were incubated with secondary antibodies [Alexa Fluor 488‐conjugated goat anti‐mouse and Alexa Fluor 555‐conjugated goat anti‐rabbit (Invitrogen)] at room temperature for 2 h. Confocal microscopy was performed to capture images (Carl Zeiss 880), which were then processed using Zen 2011 software (Carl Zeiss). Measurements were taken in a blinded manner. An average of data from five mice or dishes obtained using Image J (National Institutes of Health) was recorded.

### 2.5 2, 3, 5‐Triphenyltetrazolium hydrochloride (TTC) staining

TTC staining was conducted at 24 h after MCAO. After anesthetization with a 2% isoflurane/air mixture, the brains were immediately removed and coronally sectioned at 1 mm intervals. These sections were then incubated in 2% TTC dye (Sigma‐Aldrich). Observation and capturing images of the sections were performed with the aid of the Zeiss AxioVision 3.0 system.

### 2.6 Western blot

The tissues extracted from motor cortices and ischemic penumbra areas at different time points were lysed in precooled RAPI buffer. Total proteins were separated by 10% SDS‐PAGE gels; then, the protein in gels was transferred onto PVDF membranes. After blockage in 3% bovine serum albumin for 2 h, the membranes were incubated with the primary antibodies: (1) mouse anti‐tubulin (1:1000, ab7291; Abcam); (2) rabbit anti‐α‐Ac‐Tub (1:1000, ab179484; Abcam); (3) rabbit anti‐MEC17 (1:1000, ab184778; Abcam) overnight at 4°C; and subsequently with the secondary antibodies used were: HRP‐conjugated goat anti‐rabbit or rabbit anti‐mouse ones 1 hour at room temperature. We used the ECL Kits (Advansta) to develop membranes using imaging system (Bio‐Rad). The relative intensities of the bands were analyzed using NIH Image J software.

### 2.7 Behavioral tests

#### 2.7.1 Single‐pellet retrieval

A task was performed to assess the retrieval of a single pellet as described previously.^22^ It was performed using a clear plexiglass chamber (1 mm in thickness; with dimensions of 20 cm × 15 cm × 8.5 cm) containing a vertical slit (0.5 cm in width and 13 cm in height) positioned in the front‐facing wall of the box. Single pellet was located on a platform in front of the slits. Following a day of being habituated to the chamber by introducing a sugar pellet into the chamber, the mice were subjected to overnight food restriction before the training. The mice surpassed 90% free‐feeding weight throughout the training session. The whole process of retrieval of a single pellet by each mouse was captured from the front and the side. The success rate of retrieval was calculated as the percentage of successful retrieval / the total number of attempts to reach the pellet.

#### 2.7.2 Irregular ladder walking

In this test, mice from different groups were allowed to walk on a horizontal ladder (100 cm in length, 19 cm in height, and 10 cm in width) with irregular spacing between rungs, as per the procedure described previously.^20^, ^22^ To prevent the mice from memorizing the pattern, the pattern of irregular spacing between rungs was changed within different trials. The trials were captured in video and analyzed by blinded observers. Results were represented as a percentage of slip of contralateral limbs divided by the total steps. Baseline measurements were conducted by allowing the mice to walk on the ladder. The mice whose erroneous steps were > 10 per 50 steps were excluded.

#### 2.7.3 Accelerated rotarod test

An accelerated rotarod test was performed as previously mentioned by Yang et al.^23^ to estimate the grip strength in the mice. A gradual increase in the speed was set from 5 to 45 rpm in 2.5 min. to the time taken for the mice to fall (or cling onto and spin along with the rod for three full rotations) was recorded for statistical analysis. Three trials, separated by 10 min, were performed for each mouse.

#### 2.7.4 Cylinder test

The extent of forelimb use can be estimated using the cylinder test during spontaneous vertical exploration within a cylinder.^24^ Mice were introduced into a transparent cylinder (with a diameter of 9 cm and a height of 15 cm) that was kept on an elevated frame. A video camera was used to capture the spontaneous rearing of each mouse in the mirror and mice that made <10 contacts with the wall were not included in the analysis. The laterality index was measured using the following formula: (right forelimb usage count−left forelimb usage count) divided by (total forelimb usage count). A higher positive index is indicative of poor left hemiparesis. The experiments were performed in a blinded manner.

#### 2.7.5 Statistical analysis

Data were represented as mean ± standard error of the mean. The normality of the data was analyzed using the Shapiro–Wilk test. Two‐group comparisons were performed using the two‐tailed Student's *t*‐test. Differences among three or more groups were analyzed using one‐way ANOVA followed by Tukey's multiple comparisons. Differences among three or more groups over time were analyzed using Two‐way repeated measures ANOVA followed by Tukey's multiple comparison test. GraphPad Prism software was used. *p* < 0.05 indicated the significance level.

## 3 RESULTS

### 3.1 α‐Ac‐Tub colocalized with dendritic spines and postsynaptic protein

**FIGURE 1:**
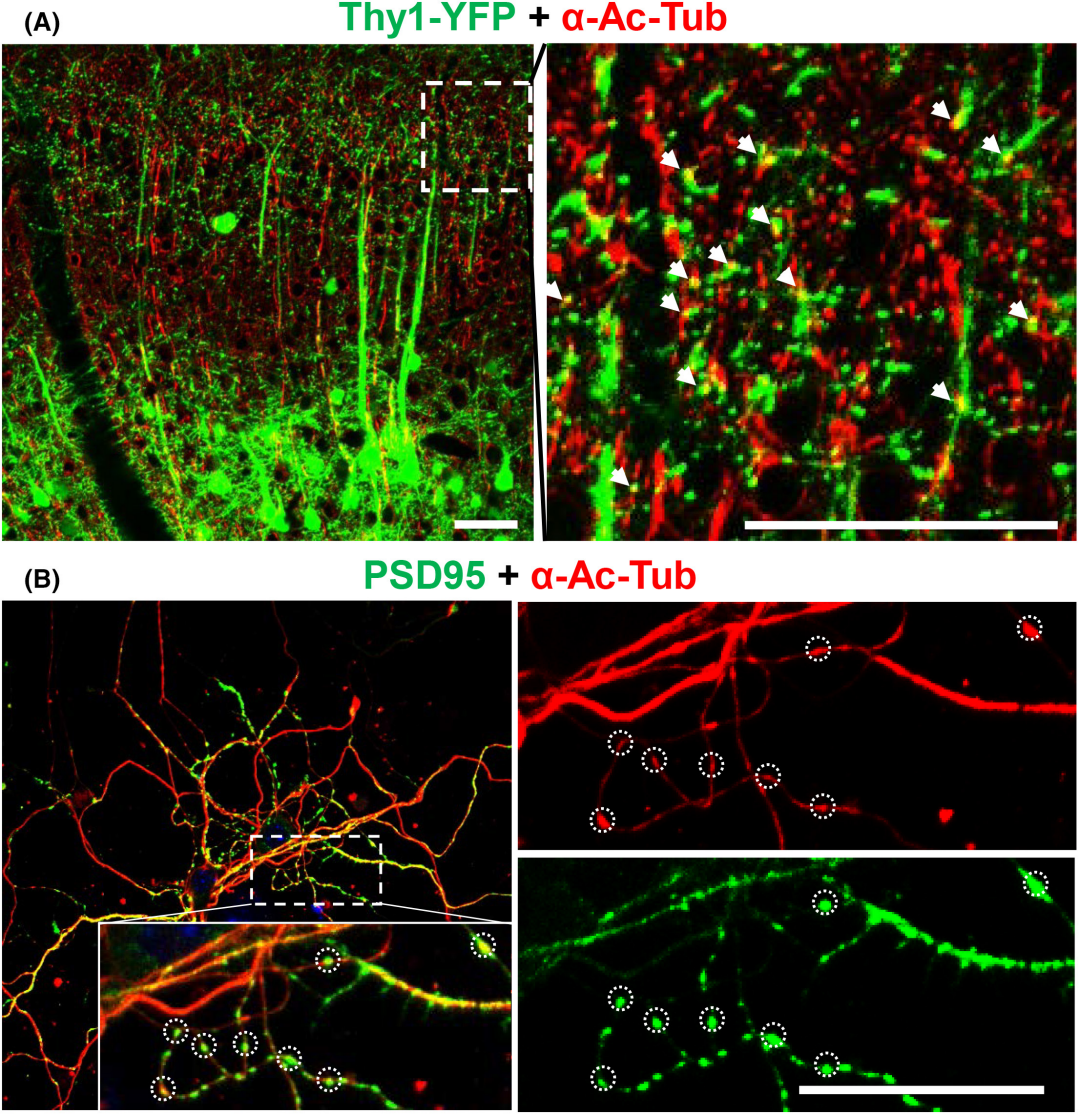
Colocalization of acetylated α‐tubulin (α‐Ac‐Tub) with the dendritic spines. (A) Representative photographs of α‐Ac‐Tub and Thy1 YFP in vivo (scale bar: 50 μm). The right picture is the magnified photograph of the white dotted square; the white arrows indicate the locations of α‐Ac‐Tub and synapses. (B) Representative photographs of α‐Ac‐Tub and PSD95 in vitro (scale bar: 20 μm). The white dotted circles indicate the colocalization points of α‐Ac‐Tub and PSD95. PSD95, postsynaptic density 95.

Thy1‐YFP mice were used to visualize the dendritic spines in vivo. The thy1‐YFP line has a strong and specific “Golgi‐like” vital marker of pyramidal neurons in layer 5 of the cerebral cortex, which produces bright fluorescence in the soma, axon, and dendritic spines. The brain sections from Thy1‐YFP mice were immunostained with α‐Ac‐Tub antibody. The results revealed that the YFP‐positive dendritic spines were colocalized with α‐Ac‐Tub (Figure 1A). Furthermore, the primary cortex neurons were extracted and cultured for 5 days to observe the dendritic spines by immunofluorescence staining of postsynaptic density 95 (PSD95), a pivotal scaffolding protein in excitatory dendritic spines. Interestingly, the PSD95‐positive dendritic spines contained α‐Ac‐Tub along the dendrites (Figure 1B).

### 3.2 The expression of MEC17 and α‐Ac‐Tub were significantly decreased after stroke in vivo and in vitro

**FIGURE 2:**
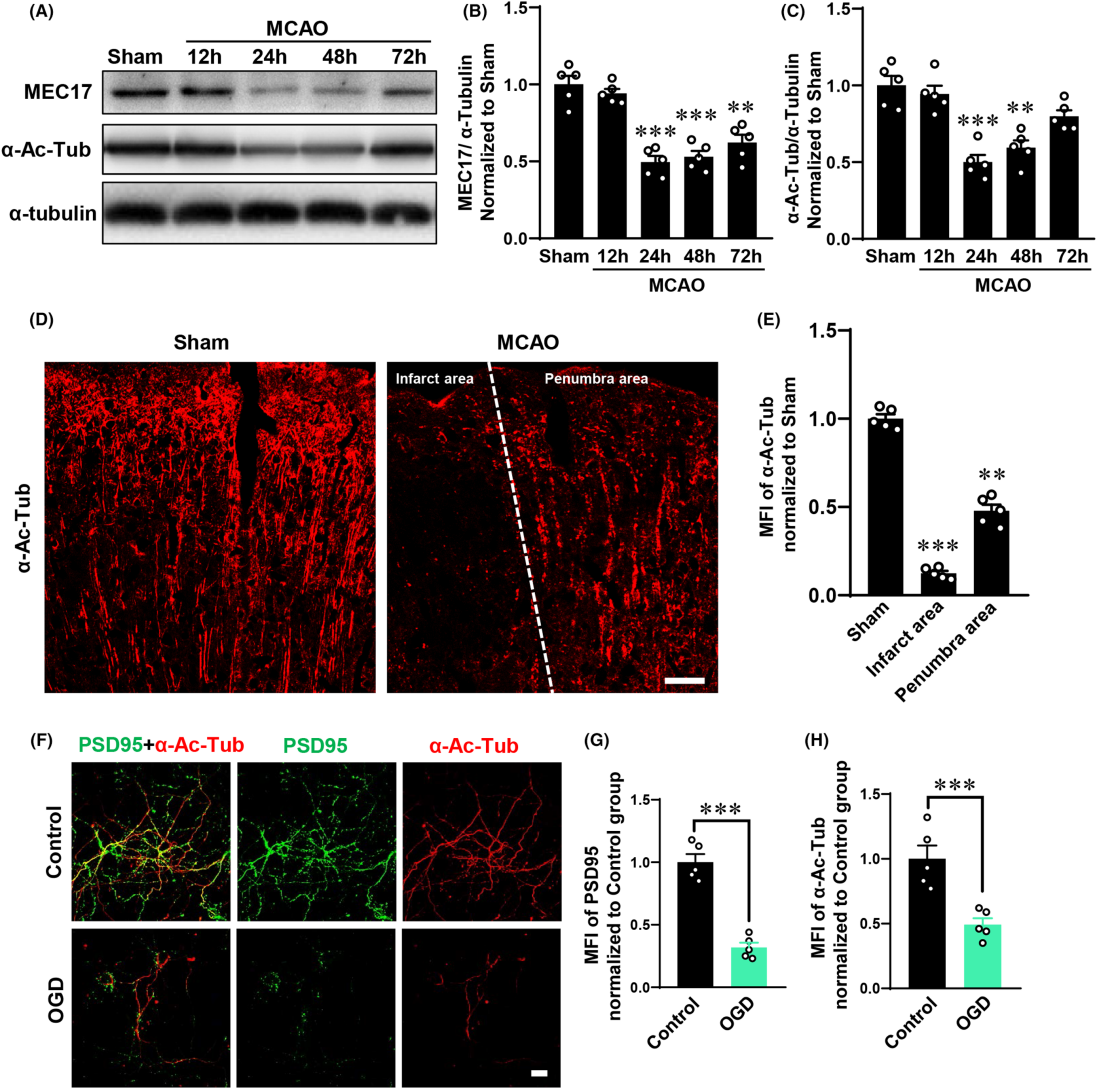
Expression of α‐Ac‐Tub and MEC17 were significantly decreased after stroke in vivo and in vitro. (A) Representative blots of MEC17, α‐Ac‐Tub, and α‐tubulin in the penumbra area at different time points after MCAO. (B‐C) The quantification of MEC17 (B) and α‐Ac‐Tub (C) in the penumbra areas at different time points after MCAO. (D) The representative photographs of α‐Ac‐Tub in each group at 24 h after MCAO (scale bar: 50 μm). (E) The MFI of α‐Ac‐Tub in different area at 24 h after MCAO. (F) Representative immunofluorescent photographs of α‐Ac‐Tub and PSD95 in vitro in the Control and OGD groups (scale bar: 25 μm). (G) The MFI of PSD95 in each group. (H) The MFI of α‐Ac‐Tub in each group. Data are represented as mean ± SEM (*n* = 5 animals for a given group or separate cell cultures). **p < 0.01 and ***p < 0.001 vs. Sham or Control group. A‐E: data were compared using one‐way ANOVA followed by Tukey's post hoc test. F‐H: data were compared using two‐tailed Student's *t*‐tests. MFI, mean fluorescence intensity. OGD, oxygen–glucose deprivation. PSD95, postsynaptic density 95.

To test the temporal change in the expression of MEC17 and α‐Ac‐Tub following cerebral ischemic stroke in vivo, we first used Western blot to examine the expression of MEC17 and α‐Ac‐Tub in the penumbra area. The expression of MEC17 and α‐Ac‐Tub were significantly decreased at 24 h and 48 h after MCAO compared with the Sham group (Figures 2A–C, *p* < 0.001). Next, the spatial change in α‐Ac‐Tub also tested by immunofluorescent staining. The mean fluorescence intensity of α‐Ac‐Tub was significantly decreased in the infarct area and penumbra area at 24 h after MCAO compared with the Sham group (Figures 2D,E, *p* < 0.01). In addition, we also investigated the expression of PSD95 and α‐Ac‐Tub in the primary cortical neurons which were subjected to OGD to mimic a stroke model. After OGD treatment, the expression of PSD95 and α‐Ac‐Tub significantly decreased compared with the Control group (Figures 2F–H, *p* < 0.001). These results indicated that α‐Ac‐Tub was decreased after stroke, which might contribute to the injury of dendritic spines.

### 3.3 α‐Ac‐Tub plays an important role in synapses protection in the penumbra area after a stroke

**FIGURE 3:**
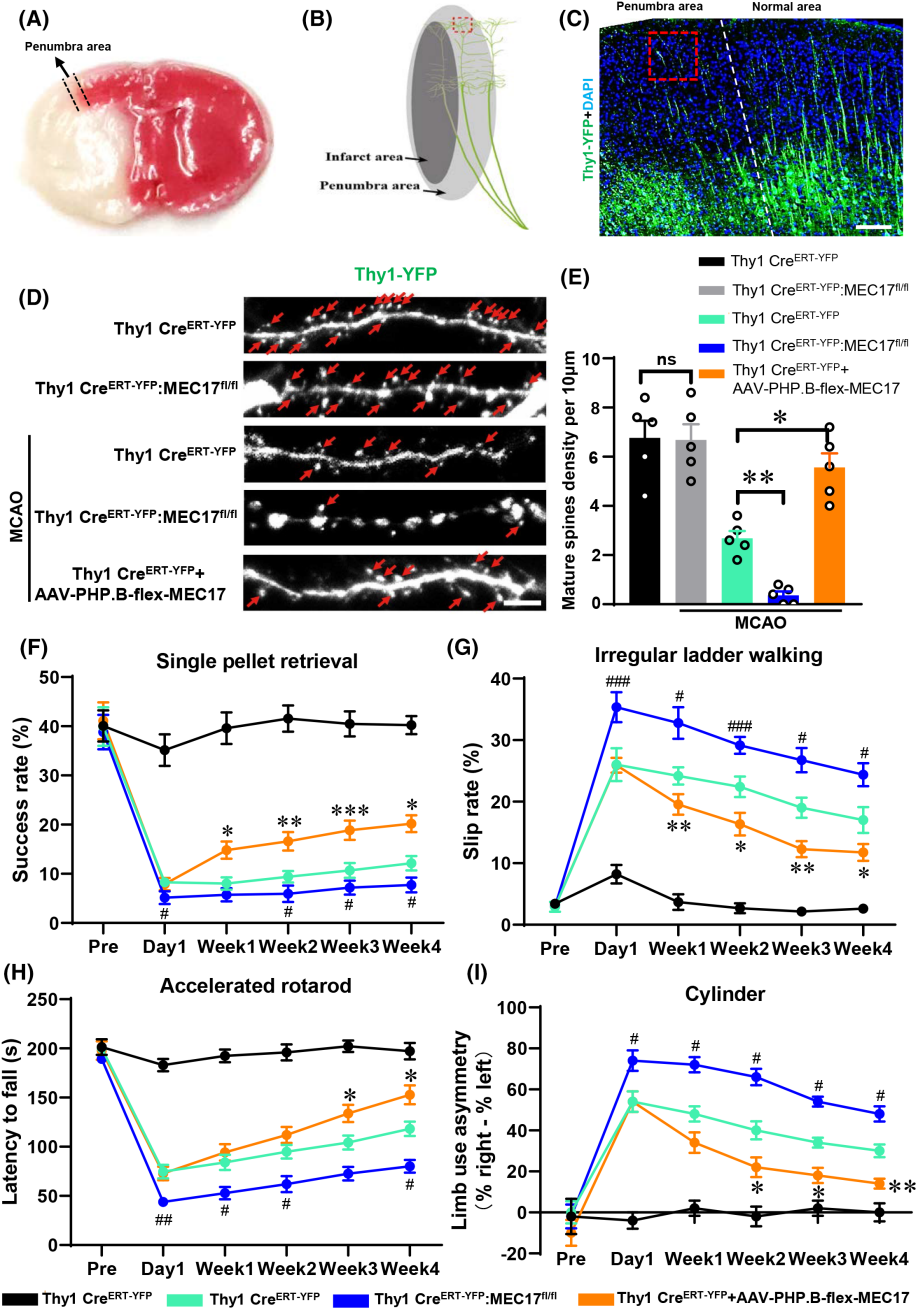
α‐Ac‐Tub plays an important role in synapses protection in the penumbra area after a stroke. (A) Representative photograph of TTC staining of the penumbra area of the stroke model. The penumbra area (pink color area between the two black dashed lines) was between the infarct core area (white color) and normal area (red color). (B) The schematic photograph of the region of interest (ROI) in the penumbra area of the stroke model; the red‐dotted square indicates the ROI. (C) The representative photograph of Thy1 YFP and DAPI (scale bar: 200 μm). The red‐dotted square is the ROI to observe the dendritic spines in the penumbra area. (D) The schematic photographs of the dendritic spines in the penumbra area in each group of Thy1 YFP mice (scale: 5 μm). Red arrows indicate the mature dendritic spines. (E) Quantitative data of the density of mature spines per 10 μm in each group. **p < 0.01, *p < 0. 05, and ns = not significant. Data were compared by performing a one‐way ANOVA followed by Tukey's post hoc test. (F) Assessment of the success rate (%) for retrieval of a single pellet after stroke. (G) Assessment of the slip rate (%) for the walking test performed on an irregular ladder after stroke. (H) Assessment of the time taken to fall (s) in an accelerated rotarod test after stroke. (I) Percentage of the limb use asymmetry in the cylinder test after stroke. F–I: ^#^ *p* < 0.05, ^##^ *p* < 0.01, and ^###^ *p* < 0.001 for MCAO + Thy1 Cre^ERT2‐YFP^ vs. MCAO + Thy1 Cre^ERT2‐YFP^::MEC17^fl/fl^; *p < 0.05, **p < 0.01, and ***p < 0.001 for MCAO + Thy1 Cre^ERT2‐YFP^ vs. MCAO + Thy1 Cre^ERT2‐YFP^ + AAV‐PHP.B‐flex‐MEC17. Two‐way ANOVA followed by Tukey's post hoc test was performed for data comparison. Data are represented as mean ± animals in each group (n = 5 animals within a single group).

The ischemic penumbra is defined as the region around the irreversibly injured core of infarction where neurons do not function but could still be potentially salvageable if appropriate treatment is received.^25^, ^26^, ^27^ Moreover, recovery is associated with the preservation of dendritic spines in the penumbra area. Therefore, we selected the penumbra area to observe the changes in dendrite spines after TBA treatment. TTC staining revealed that the penumbra area (pink color area between the two black dashed lines) was between the infarct core area (white color) and normal area (red color) (Figure 3A).^28^ Additionally, the penumbra area could be easily identified using Thy1‐YFP mice, and the red‐dotted square (Figures 3B,C) in the penumbra area was selected to observe the dendrite spines.

To specifically decrease α‐Ac‐Tub expression in the pyramidal neurons, Thy1 Cre^ERT2‐eYFP^ mice and MEC17^fl/fl^ mice were mated to produce Thy1 Cre^ERT2‐eYFP^::MEC17^fl/fl^ mice. The expression of α‐Ac‐Tub in the motor cortex from Thy1 Cre^ERT2‐eYFP^::MEC17^fl/fl^ mice was decreased to 64.3% of the Thy1 Cre^ERT2‐eYFP^ mice (Figure S1A,C). Moreover, the AAV‐PHP.B‐DIO‐MEC17 virus was intravenously injected into Thy1 Cre^ERT2‐eYFP^ mice to upregulate MEC17 and α‐Ac‐Tub in the pyramidal neurons. The expression of α‐Ac‐Tub in the motor cortex was increased to 142.3% of the Thy1 Cre^ERT2‐eYFP^ mice after AAV‐PHP.B‐DIO‐MEC17 injection (Figure S1A,B). Interestingly, MEC17 knockout in the pyramidal cells did not affect the density of mushroom‐shaped mature dendritic spines (Figure 3D,E; *p* > 0.05) but aggravated the injury to dendritic spines after stroke compared with the MCAO + Thy1 Cre^ERT2‐eYFP^ group (Figure 3D,E; *p* < 0.01). In contrast, MEC17 overexpression significantly rescued the injury to dendritic spines after stroke compared with the MCAO + Thy1 Cre^ERT2‐eYFP^ group (Figure 3D,E; *p* < 0.05).

To assess whether TBA treatment could promote recovery after stroke, the mice were subjected to several behavioral tests related to skilled motor functions, namely, single‐pellet reaching, irregular ladder walking, rotarod, and cylinder. MEC17 knockout in the pyramidal neurons aggravated the behavioral dysfunction compared with the MCAO + Thy1 Cre^ERT2‐eYFP^ group from Day 1 to 4 weeks after stroke (Figure 3F–I). Conversely, MEC17 overexpression in the pyramidal neurons significantly alleviated the motor dysfunction after stroke compared with the MCAO + Thy1 Cre^ERT2‐eYFP^ group (Figure 3F–I). Collectively, these results suggested that the preserved mature dendritic spines functionally mediated better behavioral performance.

### 3.4 TBA treatment alleviated the injury of dendritic spines in neurons with OGD

**FIGURE 4:**
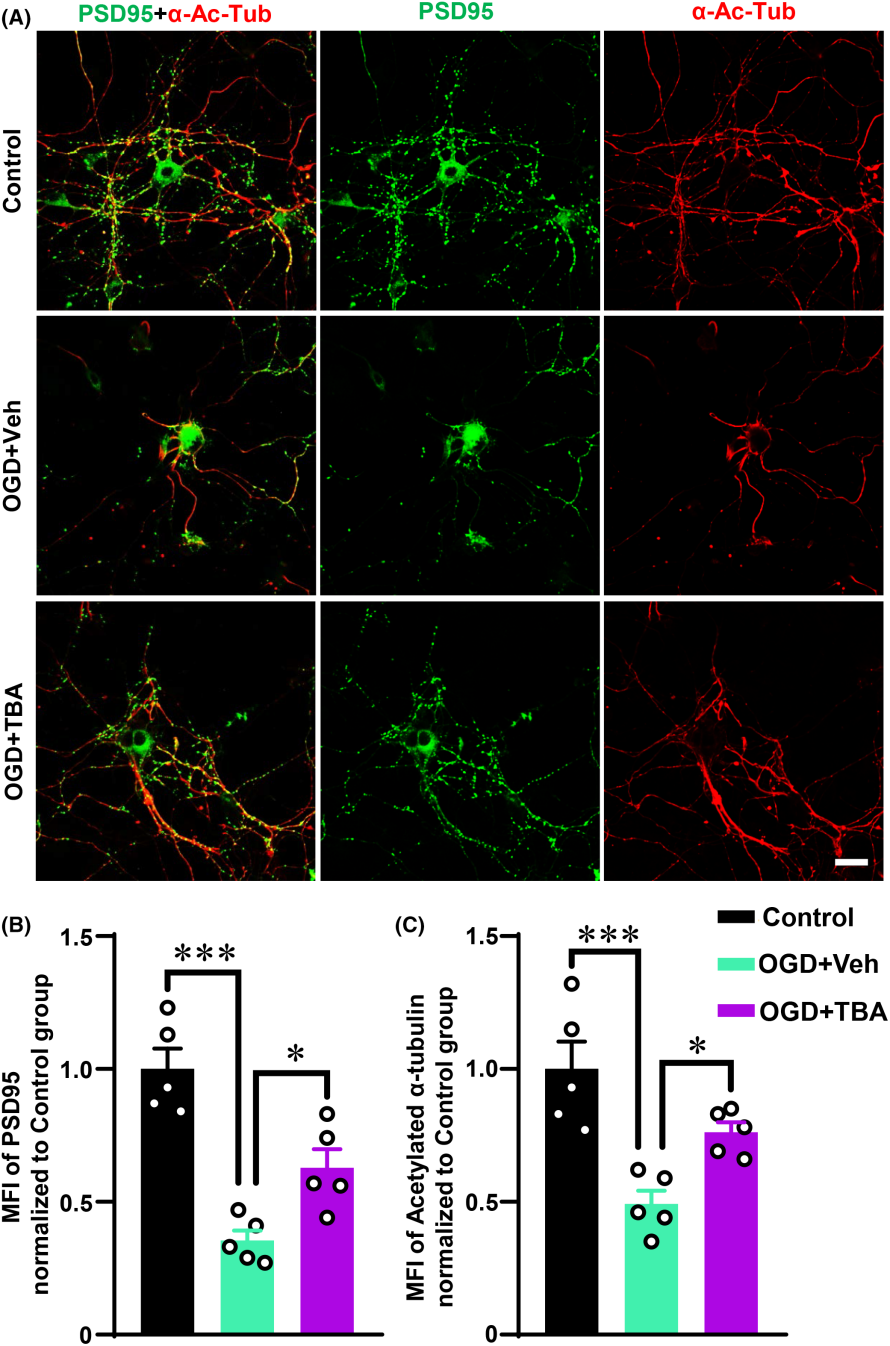
TBA alleviated synapse injury after OGD. (A) Representative immunofluorescent photographs of α‐Ac‐Tub and PSD95 in vitro in each group after OGD and TBA treatment (scale bar: 25 μm). (B) The MFI of PSD95 in each group. (C) The MFI of α‐Ac‐Tub in each group. Data are represented as mean ± SEM (n = 5 separate cell cultures). *p < 0.05 and ***p < 0.001. Data were compared using one‐way ANOVA followed by Tukey's post hoc test. MFI, mean fluorescence intensity. OGD, oxygen–glucose deprivation. PSD95, postsynaptic density 95. TBA, tubastatin A.

HDAC6 is the major deacetylase of α‐Ac‐Tub and HDAC6 inhibitors could significantly increase α‐Ac‐Tub expression.^29^ TBA, an HDAC6 inhibitor, was used to treat the primary neurons to upregulate α‐Ac‐Tub expression as described in our study as well as other previous studies.^20^, ^30^, ^31^, ^32^ Interestingly, TBA treatment could rescue the loss of PSD95 and α‐Ac‐Tub compared with that in the OGD + Veh group (Figure 4A–C, *p* < 0.05). This indicated that the increased expression of α‐Ac‐Tub alleviated OGD‐induced injury to dendrite spines.

### 3.5 TBA protected the dendritic spines in the penumbra area and alleviated motor dysfunction after the stroke

**FIGURE 5:**
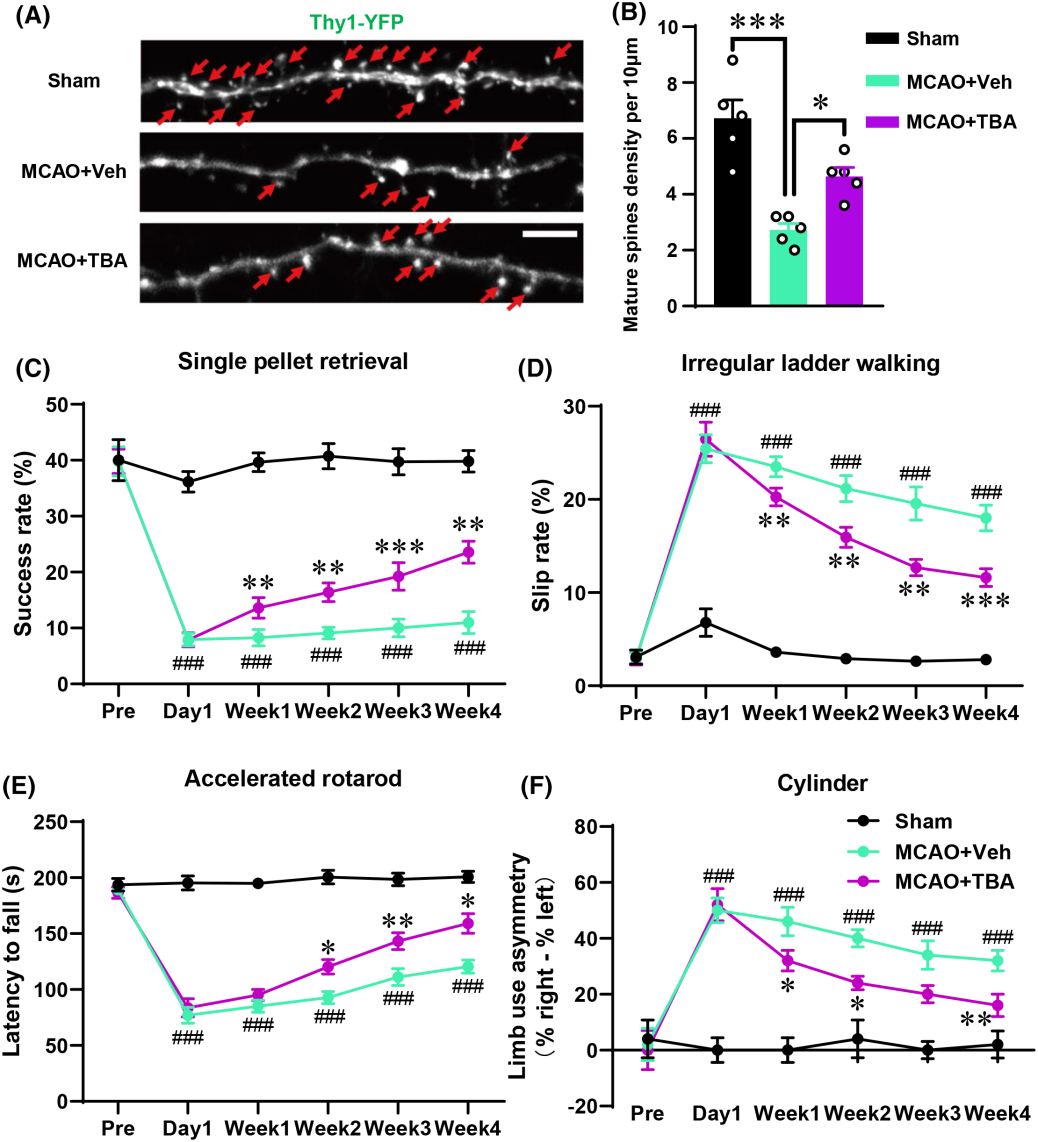
TBA‐alleviated synapses injury and alleviated motor dysfunction after the stroke. (A) Schematic photographs for the dendritic spines in the penumbra area in each group of Thy1 YFP mice (scale bar: 5 μm). Red arrows indicate the mature dendritic spines. (B) Quantitative data of the density of mature spines per 10 μm for a given group. *p < 0.05 and ***p < 0.001. Data were compared using one‐way ANOVA followed by Tukey's post hoc test. (C) Assessment of the success rate (%) in the test performed to retrieve a single pellet after stroke. (D) Assessment of the slip rate (%) for the irregular ladder walking test after stroke. (E) Assessment of time taken to fall(s) in the accelerated rotarod test after stroke. (F) Percentage of the limb use asymmetry in the cylinder test after stroke. C–F: ^###^ *p* < 0.001 for Sham vs. MCAO + Veh groups; ***p < 0.001, **p < 0.01, and *p < 0.05 for MCAO + Veh vs. MCAO + TBA group. Data were compared using a two‐way ANOVA followed by Tukey's post hoc test. Data are represented as mean ± SEM (*n* = 5 animals for a given group). TBA, tubastatin A.

The MCAO mice were treated with TBA to investigate the protection effects of α‐Ac‐Tub after stroke. Similarly, the number of mature dendritic spines was significantly decreased in the MCAO + Veh group than in the Sham group (Figures 5A,B; *p* < 0.001). In contrast, a significant increase was observed in the number of mature spines in the TBA‐treated group (Figures 5A,B; *p* < 0.05). Behaviorally, the MCAO + Veh group exhibited significant impairments in above behavioral tests from day 1 to 4 weeks (Figures 5C–F; *p* < 0.001). However, compared with that of the MCAO + Veh group, TBA treatment significantly increased the success rate of pellet retrieval (Figure 4C), decreased slip ratio of contralateral limbs in the irregular ladder walking test (Figure 4D), increased the latency to fall in the accelerated rotarod test (Figure 4E), and showed a decrease in the use of right forelimb in the cylinder test (Figure 4F) after stroke. Collectively, our data indicated that TBA‐induced upregulation of α‐Ac‐Tub protected mature dendritic spines and alleviated behavioral dysfunction after stroke.

## 4 DISCUSSION

**FIGURE 6:**
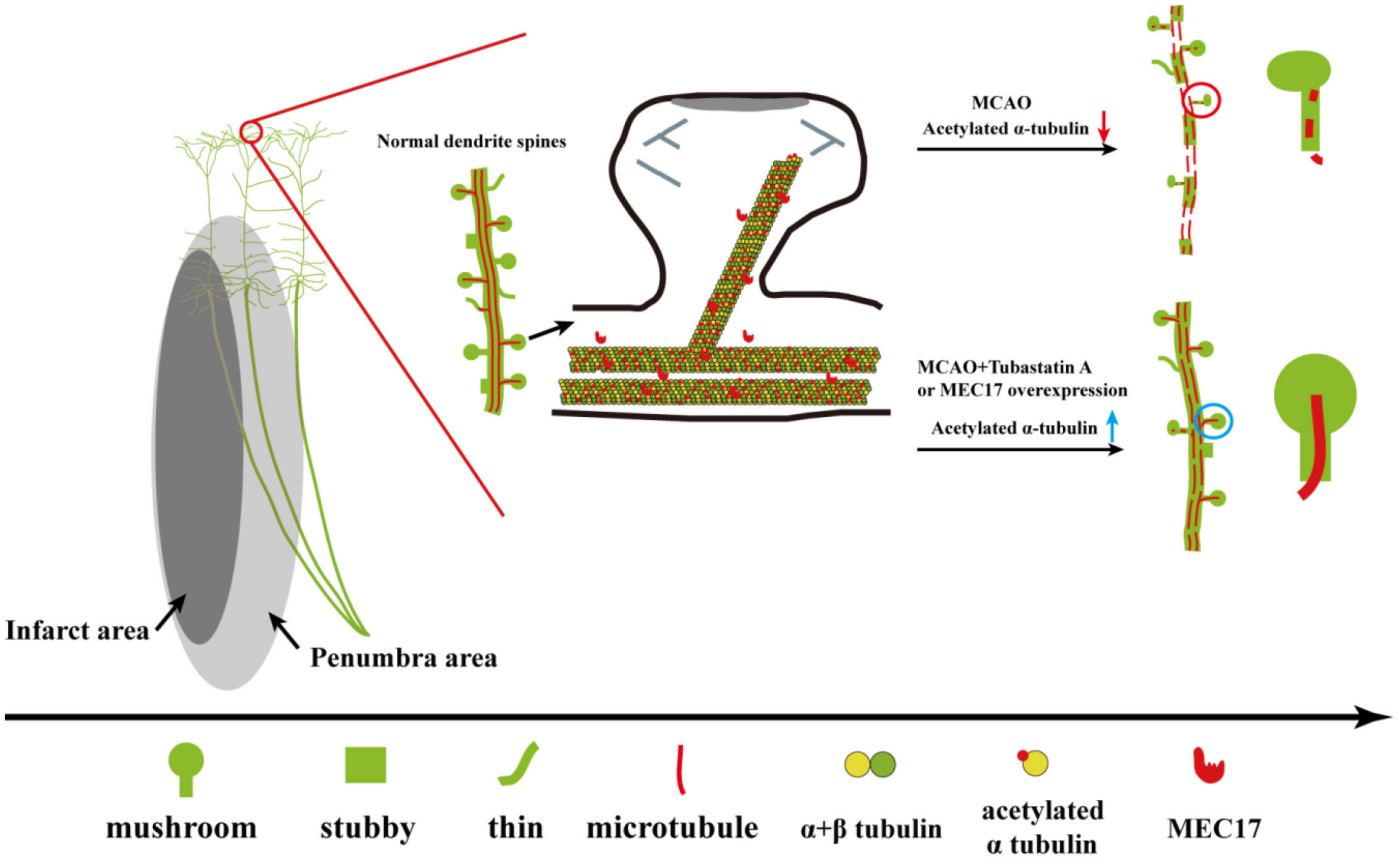
Schematic summary of this study. α‐Ac‐Tub plays a crucial role in maintaining the structure and function of dendritic spines. Synapse injury leads to the disruption of the neural circuit and neurological dysfunction after stroke. Protection of more dendritic spines in the penumbra area is a promising therapeutic strategy for stroke. α‐Ac‐Tub was colocalized with postsynaptic density protein. Specifically, the knockout of MEC17 (an α‐tubulin acetyltransferase) could aggravate the synapse injury of pyramidal neurons in the penumbra area. However, specific upregulation of MEC17 in the pyramidal neurons alleviated synapse injury in the penumbra area and relieved motor dysfunction after stroke. Moreover, TBA treatment alleviated synapse injury in the penumbra area and relieved motor dysfunction after stroke.

Primary findings of the study include the following: (1) The α‐Ac‐Tub was colocalized with postsynaptic protein PSD95. (2) Although knockout of MEC17 in the pyramidal neurons did not affect the density of dendritic spines, it significantly aggravated injury to dendritic spines in the penumbra area and motor dysfunction after stroke. (3) MEC17 overexpression and TBA treatment could protect the dendritic spines and alleviate motor dysfunction after stroke (Figure 6). Therefore, protection of more dendritic spines in the penumbra area is a promising therapeutic strategy for stroke.

Recovery after cerebral ischemia has been attributed to plasticity and circuit reorganization in the brain.^33^, ^34^ Although poststroke plasticity events can be controlled by neurorehabilitative practices,^4^, ^35^ drugs,^22^ brain stimulation,^36^ and sensory inputs^24^ enhanced recovery in animal models. The action of these manipulations is related to plasticity in the brain circuits but limited processes of functional recovery. Interestingly, a previous study reported that dendritic structure can be recovered after reperfusion restricted to a relatively small penumbra region. These preserved dendritic spines exhibited remarkable flexibility and began to process sensory stimuli from multiple limbs as remapping proceeded.^37^ In our study, we reported that functional recovery is associated with the preservation of the dendritic spines, particularly the mature ones, in the penumbra area. Our finding suggested that preventing injury to dendritic spines at the early stage of stroke is an additional promising therapeutic strategy for functional improvement after stroke.

Dendritic spines are small, specialized, and thin protrusions arising from neurons where excitatory synapses are at their maximum. Dendritic protrusions are divided into four classes based on their morphology: mushroom (mature spines), stubby (mature spines), thin (immature spines), and filopodia‐like (immature spines).^38^ In adults, a mature synaptic contact is present in the majority of the spines, whereas 20% are immature.^39^ A previous study demonstrated that the mature spines are memory spines and integrate into the neural circuits for specific functions. Loss of these mature spines indicates disruption of neural circuits.^34^ In this study, significant loss of mature spines was observed in the ischemic penumbra accompanied by impaired skilled functions, such as pellet retrieval by forepaw. Protection of the mature spines by upregulation of α‐Ac‐Tub using pharmacological and genetic methods could alleviate motor dysfunction after stroke. Mechanically, PSD‐95 recruitment plays a crucial role in synaptic maturation and stabilization of the spine. Interestingly, a previous in vivo study reported that approximately 20% of spines are devoid of fluorescence‐tagged PSD‐95 and are short‐lived.^40^ These spines might be immature or filopodia‐like. Furthermore, we demonstrated that upregulation of α‐Ac‐Tub recused the loss of PSD95 after OGD treatment. These results provided insights for alleviating neurological dysfunction by protecting mature spines after stroke.

Cytoskeletal components play a crucial role in the morphology and functions of dendritic spines. Actin and actin‐associated have been shown to regulate spine morphology and associated dynamics involved in activity‐dependent plasticity.^41^ However, > 80% of spines contain a mature synaptic contact and only 5% of spines undergo formation and elimination in adults.^39^ Therefore, a more stable cytoskeletal component is needed for the structural and functional maintenance of mature spines. Previous studies reported that polymerized MTs enter mature dendritic spines and are associated with their enlargement and stabilization.^7^, ^8^, ^42^ Interestingly, α‐Ac‐Tub is most abundant in polymerized MTs.^16^ Moreover, a previous study demonstrated that cylindromatosis tumor suppressor protein (CYLD) regulates dendritic growth and postsynaptic differentiation in cultured hippocampal neurons which was mediated by the α‐Ac‐Tub.^43^ This indicates that α‐Ac‐Tub could enter the mature spines and play important role in maintaining the structure of the dendritic spines. Consistently, we further observed that α‐Ac‐Tub was colocalized with PSD95 and could recuse the PSD95 loss after OGD. Additionally, α‐Ac‐Tub upregulation using pharmacological and genetic methods could preserve the mature dendritic spines in the penumbra area and alleviate motor dysfunction after stroke. Furthermore, although α‐Ac‐Tub downregulation in the sensory neurons led to profound deficits in detecting mechanical stimuli,^44^ it did not affect the density of mature spines in the cortex.

There are several limitations of our study. Firstly, α‐tubulin is not the only substrate protein of HDAC6. To specifically investigate the effect of K40 α‐Ac‐Tub in the dendritic spines, gene site‐directed mutation is needed to manipulate the acetylation on lysine 40 of α‐tubulin in further work. Secondly, MTs contain numerous PTMs, including detyrosination, acetylation, polyglutamylation, and polyglycylation^12^ This study was limited to just understanding the function of α‐Ac‐Tub in dendritic spines after stroke. More studies are needed to understand the significance of other PTMs in dendritic spines. Thirdly, calcium imaging using two‐photon is worth investigating to visualize the function of the protected dendritic spines in vivo.

## 5 CONCLUSION

This study suggested that α‐Ac‐Tub plays a crucial and direct role in the maintenance of morphology and functions of dendritic spines after stroke. Moreover, α‐Ac‐Tub is a novel therapeutic target for preventing injury to dendritic spines in the penumbra area and subsequent functional impairment after stroke.

## AUTHORS' CONTRIBUTIONS

The study was planned and designed by TC and HF. Data acquisition and analysis were performed by CY, XC, XL, YL, CZ, and YW. YY and TC helped in drafting a substantial part of the manuscript. Critical revisions of the manuscript were performed by YW, YY, TC, and HF. The manuscript has been reviewed and subjected to permission for publication by all the included authors.

## FUNDING INFORMATION

This study was funded and supported by the National Natural Science Foundation of China (81901267), Chongqing Postdoctoral Innovative Talent Support Program (CQBX2021008), Natural Science Foundation of Jiangsu Province (BK20221206), Young Elite Scientists Sponsorship Program of Jiangsu Province (TJ‐2022‐028), and China Postdoctoral Science Foundation (2022 M723868).

## CONFLICT OF INTEREST STATEMENT

The authors hereby declare that there is no conflict of interest.

## ETHICS APPROVAL

All the in vivo protocols were approved by the Ethics Committee of the Army Medical University (AMUWEC20210680). Experiments performed in this study followed the guidelines provided by Animal Research standards: Reporting of In Vivo Experiments (ARRIVE).

## Supporting information


Figure S1:

## Data Availability

The data produced and analyzed in this study are readily available in the final draft of the manuscript. Used datasets can be retrieved from the corresponding author.
