# Supplementary material for: Acetylated α‐tubulin alleviates injury to the dendritic spines after ischemic stroke in mice
Source: CNS Neurosci Ther. 2023 Mar 25;29(8):2327–38. doi: 10.1111/cns.14184 (PMC10352872; doi:10.1111/cns.14184)
Supplement: Supplementary file 1 — Figure S1: [file CNS-29-2327-s001.docx]

**Supplementary figure: The expression of α-Ac-Tub after overexpression or knockout of MEC17.**


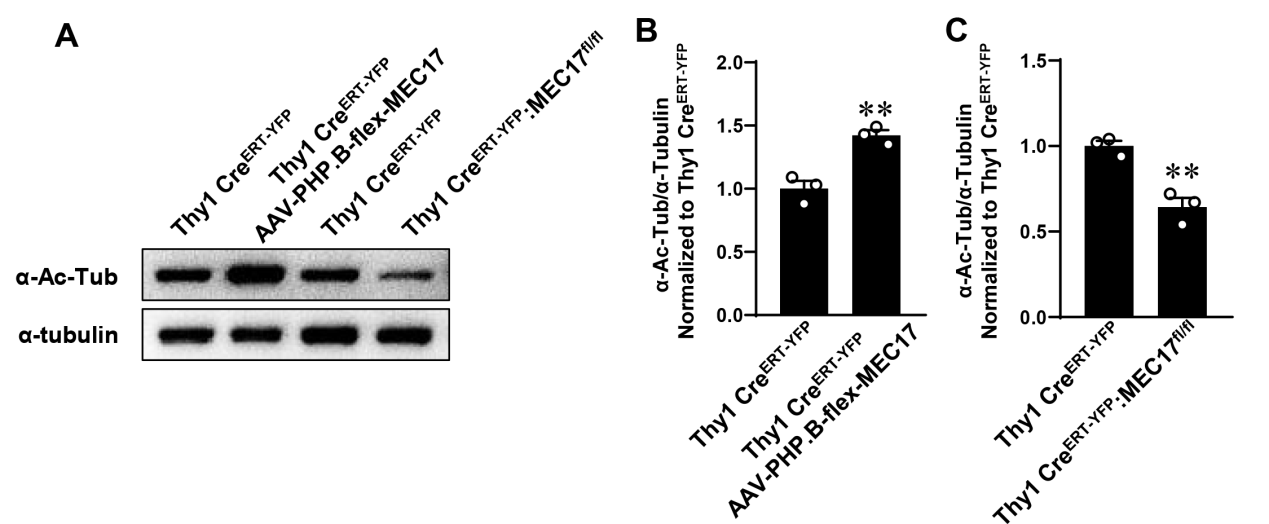


**(A)** Representative blots of α-Ac-Tub and α-tubulin in the motor cortices in each group. **(B)** The quantification of α-Ac-Tub in the groups of Thy1 Cre^ERT2-eYFP^ and Thy1 Cre^ERT2-eYFP^+AAV-PHP.B-DIO-MEC17 mice. **(C)** The quantification of α-Ac-Tub in the groups of Thy1 Cre^ERT2-eYFP^ and Thy1 Cre^ERT2-eYFP^::MEC17^fl/fl^ mice. Data are represented as mean ± SEM (n = 3 animals for a given group). ***P* < 0.01 *vs.* Thy1 Cre^ERT2-eYFP^ group. Data were compared using two-tailed Student's *t*-tests.
